# Stress Induces Endotoxemia and Low-Grade Inflammation by Increasing Barrier Permeability

**DOI:** 10.3389/fimmu.2015.00223

**Published:** 2015-05-15

**Authors:** Karin de Punder, Leo Pruimboom

**Affiliations:** ^1^Institute of Medical Psychology, Charité University Medicine, Berlin, Germany; ^2^Natura Foundation, Numansdorp, Netherlands

**Keywords:** endotoxemia, endotoxin, inflammation, intestinal permeability, lipopolysaccharide, stress, tight junction

## Abstract

Chronic non-communicable diseases (NCDs) are the leading causes of work absence, disability, and mortality worldwide. Most of these diseases are associated with low-grade inflammation. Here, we hypothesize that stresses (defined as homeostatic disturbances) can induce low-grade inflammation by increasing the availability of water, sodium, and energy-rich substances to meet the increased metabolic demand induced by the stressor. One way of triggering low-grade inflammation is by increasing intestinal barrier permeability through activation of various components of the stress system. Although beneficial to meet the demands necessary during stress, increased intestinal barrier permeability also raises the possibility of the translocation of bacteria and their toxins across the intestinal lumen into the blood circulation. In combination with modern life-style factors, the increase in bacteria/bacterial toxin translocation arising from a more permeable intestinal wall causes a low-grade inflammatory state. We support this hypothesis with numerous studies finding associations with NCDs and markers of endotoxemia, suggesting that this process plays a pivotal and perhaps even a causal role in the development of low-grade inflammation and its related diseases.

## Introduction

Inflammation is the response of the innate immune system triggered by stimuli like microbial pathogens and injury. Acute systemic inflammation such as in sepsis, trauma, burns, and surgery is characterized by a quick increase in plasma levels (up to 100-fold) of pro-inflammatory cytokines and acute phase proteins, while in low-grade inflammation, there is a sustained but only two to threefold increase in circulation pro-inflammatory mediators (1). Chronic low-grade inflammation is characteristic for many non-communicable diseases (NCDs) including diabetes type II, cardiovascular disorders, autoimmune diseases, chronic fatigue syndrome, depression, and neurodegenerative pathologies, but until now the exact mechanism behind the elevated levels of inflammatory mediators found in these conditions is not well understood (2–5).

Inflammation can be induced by the binding of pathogen-associated molecular patterns (PAMPs) to toll-like receptors (TLRs), which are expressed on different cells types including immune cells, adipocytes, and endothelial cells. The most extensively studied PAMP is lipopolysaccharide (LPS) or endotoxin (the terms LPS and endotoxin will be used interchangeably throughout the rest of the article), a major cell wall component of Gram-negative bacteria, which is normally present in the human circulation in very low concentrations. It has been hypothesized that most of this circulating LPS is derived from the gut, since the gut-microbiota is the biggest source of Gram-negative bacteria-derived LPS. However, LPS found in the circulation could also be derived from Gram-negative bacteria residing in the oral cavity, respiratory, and genitourinary tracts, or can be food-derived (6–8). Under certain circumstances, there can be an increase of endotoxin translocation across the intestinal barrier, leading to mildly increased concentrations in the blood circulation. This process has been associated with several NCDs, like depression (9), chronic fatigue syndrome (10), chronic heart failure (11), type 2 diabetes (12), autism (13), non-alcoholic fatty liver disease (NAFLD) (14), and inflammatory bowel disease (IBD) (15), diseases that are all linked to chronic systemic low-grade inflammation, indicating that endotoxemia could be an important contributor in the development of these conditions.

Here, we hypothesize that stress-induction leads to a more permeable intestinal wall intended to facilitate an increase in the availability of water, sodium, and energy-rich substances necessary to meet the increased metabolic demand induced by the stressor. Modern life-style factors, such as long-term psychosocial stress and components of our “Western” diet constantly challenge the stress-axis and further compromise intestinal barrier function, resulting in endotoxemia, low-grade inflammation, and its related diseases. We support our hypothesis by describing literature surrounding stress- and immune system-activation processes and their relation to gut barrier function and explain how life-style choices impact all these systems. In addition, we present a vast amount of literature describing associations with NCDs and markers of endotoxemia. Overall, we conclude that stress-induced disrupted barrier function in parallel with elevated circulating endotoxin levels may underlie disease onset and progression and should be considered much more than just a risk factor for chronic disease; it could be a cause.

## Bacterial Toxins Activate the Immune System via TLRs

Lipopolysaccharide, the major cell wall component of Gram-negative bacteria, is characterized by its capacity to induce inflammation, fever, shock, and death (1). Additionally in recent years, other cell wall components of Gram-negative and -positive bacteria have been recognized to have endotoxic properties (16), but these will not be further addressed in the rest of the paper. Endotoxins are released from bacteria during infection or as a consequence of bacterial lysis. Although both whole bacteria and bacterial toxins can translocate transcellular or paracellular into the lymph, blood, and mesenteric lymph nodes, it is still not precisely clear if the presence of endotoxin in the blood circulation (endotoxemia) also presents whole bacteria translocation across the intestinal wall (17).

Inflammation can be induced by the binding of LPS to TLR4. The lipid-A moiety of LPS interacts with the LPS-sensing machinery composed of TLR4, myeloid differential protein 2, CD14, and LPS-binding protein (LBP). LBP transports and delivers circulating aggregates of LPS to lipoproteins, resulting in hepatic clearance, or delivers LPS to CD14 (the membrane-bound or secreted, soluble form of this molecule), leading to TLR4 activation. TLR4 activation activates two transcription factors, activator protein (AP)-1 and nuclear factor κB (NF-κB) (18, 19), and stimulates the production of pro-inflammatory mediators such as prostaglandin 2 (PGE2) (20), tumor necrosis factor (TNF)-α, interleukin (IL)-1β, IL-6, interferon (IFN)-γ, and the acute phase protein, C-reactive protein (CRP) (19). Simultaneously, an uncontrolled pro-inflammatory reaction is prevented by the induction of TLR4, NF-κB, and AP-1 signaling inhibitors, which are probably involved in creating endotoxin tolerance (21). LPS tolerance is defined as a reduced responsiveness to a LPS challenge following a first encounter of endotoxin (22). It has been suggested that the dose of LPS exposure is important for determining the switch between LPS tolerance and priming. For example, in macrophages, high LPS concentrations induced a robust pro-inflammatory response in parallel with the activation of inhibitory feedback mechanisms. Lower concentrations of LPS, like those observed in NCDs, removed transcriptional suppressors on the promoters of pro-inflammatory genes and induced a mild but persistent expression of pro-inflammatory mediators (21, 23).

## Intestinal Barrier Function

### The paracellular pathway is important for water, mineral, and nutrient uptake

The intestinal barrier allows for the regulated uptake of water, minerals, and nutrients and protects the gut lumen from damage due to harmful substances. Components can cross the epithelial barrier by active transport and endocytosis (transcellular) or via the paracellular route. Because hydrophilic solutes are limited to cross lipid membranes of epithelial cells, the paracellular route is an important and major route for the transport of water, solutes, and minerals across the intestinal barrier (24, 25). Active glucose, sodium, and water uptake is mediated by the activity of sodium-dependent glucose co-transporters (SGLTs) (26). The transcellular absorption of glucose and sodium and the resulting basolateral disposition of glucose and sodium by these transporters opens up the paracellular pathway structure and allows the passive flow of water and small nutrients by creating an osmotic gradient (27).

**Figure 1:**
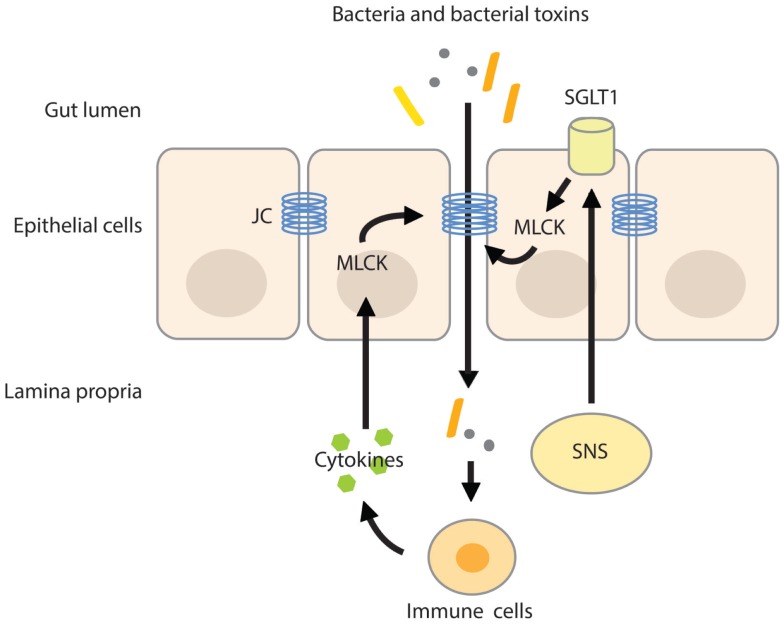
**MLC phosphorylation increases intestinal permeability**. Activation of the SNS increases intestinal permeability by stimulating the activity of SGLT1 on epithelial cells. Activation of SGLT1 is paralleled by MLC phosphorylation by MLCK, inducing actomyosin contraction and reorganization of the tight junction. The resulting increase in paracellular permeability raises the possibility of translocation of bacteria and/or their toxins across the more permeable gut barrier. Pro-inflammatory cytokines produced by activated immune cells residing in the lamina propria further increase intestinal permeability by activating MLCK. JC, junctional complex.

Intestinal permeability is a measure of the barrier function of the gut and relates to the paracellular space surrounding the brush border surface of the enterocytes and the junctional complexes (28). The junctional complex, containing tight junctions, adherens junctions, and desmosomes is an important regulator of the paracellular pathway and allows the passage of water, solutes, and ions, but under normal conditions provides a barrier to larger molecules (28, 29). The claudin family of junctional transmembrane proteins has a substantial effect on paracellular permeability. While one group of sealing claudins makes the paracellular barrier less permeable, the other group of claudins is known to increase paracellular permeability by the formation of pores that increase permeability for small solutes (30, 31). The expression of claudin proteins varies between tissues, explaining the variances in permeability of tight junctions among tissues (27). The paracellular pathway can be divided into the pore and non-pore pathway. The pore pathway is mainly controlled by the expression of claudins, while the non-pore pathway is more sensitive to cytoskeletal disruptions (30). Cytoskeletal rearrangements can be induced by phosphorylation of the regulatory myosin light chain (MLC), induced by MLC-kinase (MLCK). Phosphorylation of the MLC facilitates myosin binding to actin and therefore aids in cytoskeletal contractility. MLCK can be activated by cytokines such as TNF-α, causing increases in tight junction permeability by actomyosin contraction and reorganization of the tight junction (32, 33). In addition, SGLT1 activation and associated increases in tight junction permeability are also paralleled with phosphorylation of MLC, indicating that MLCK is an important mediator in tight junction and paracellular permeability regulation (25, 34) (Figure 1).

Increased intestinal permeability has been associated with autoimmune diseases, such as type 1 diabetes (35), rheumatoid arthritis, multiple sclerosis (36), and diseases related to chronic inflammation, like IBD (36, 37), asthma (38), chronic fatigue syndrome, and depression (10, 39). It has been hypothesized that chronic intestinal hyper-permeability results in a pro-inflammatory phenotype induced by the enhanced paracellular translocation of microbial (and dietary) antigens across the gut barrier (40).

## Stress Increases Permeability of the Intestinal Barrier

Stressful stimuli activate the sympathetic nervous system (SNS) and hypothalamic–pituitary–adrenal (HPA)-axis. Activation of both systems increases the availability of water, minerals, and energy-rich substances in order to meet with the body’s metabolic demand (41, 42). The SNS responds instantly to physical and psychological stress by reallocating energy into different organs by neuronal regulation of heart rate, blood flow, release of catecholamines (adrenalin and noradrenalin) from the adrenal medulla (43), and stimulation of the renin–angiotensin–aldosterone system (44), involved in retention of water and sodium from the kidneys. In addition to the kidneys, water and sodium reabsorption can also be achieved at the level of the intestine. The intestinal wall is innervated by adrenergic sympathetic nerve fibers that upon stimulation increase water and sodium absorption (45, 46), which is paralleled by increases in intestinal permeability. The SNS-induced increase in permeability is likely mediated by β2-adrenergic receptors expressed on epithelial cells (47). Activation of the β2-adrenergic-receptors stimulated SGLT1-mediated glucose absorption from the gut (48, 49) and the resulting basolateral disposition of glucose and sodium by these transporters opens up the paracellular pathway (27) (Figure 1). Not surprisingly, blockage of the SNS by means of thoracic epidural anesthesia resulted in the blockage of the endotoxin-induced increase in intestinal permeability in rats (50).

Activation of the HPA-axis leads to the release of glucocorticoids that potentiate some of the actions of catecholamines. Essential to this response are the neurons in the paraventricular nucleus of the hypothalamus expressing corticotropin-releasing hormone (CRH) and other co-secretagogues, such as arginine vasopressin (AVP) and oxytocin, both involved in the regulation of water homeostasis. AVP and CRH trigger the immediate release of adrenocorticotropic hormone (ACTH) from the anterior pituitary, which in turn induces the release of glucocorticoids and to some extend mineralocorticoids from the adrenal cortex, stimulating gluconeogenesis and increasing sodium and water retention, respectively (51, 52). Intestinal permeability is regulated by several components of the HPA-axis.

In epithelial HT-29 monolayers, exposure to CRH resulted in an increased response to LPS as reflected by a decrease in transepithelial resistance and a significant increase in the expression of the pore forming protein, claudin-2. Interestingly enough, these effects were mediated by an increase in TLR4 expression, an observation that could be repeated in mice treated with the water-avoid stressor (53). TLR4 activation resulted in the activation of the transcription factor NF-κB, which has specific binding sites in the claudin-2 gene promoter (54), indicating that in epithelial cells CRH affects both intestinal permeability and inflammatory pathways.

In rats, exposure to restricted stress or swimming stress increased intestinal permeability throughout the whole intestinal tract as measured by the fractional secretion of the urinary recovery of sucrose (reflecting gastric permeability), the lactulose–mannitol ratio (as a marker for small intestinal permeability), and sucralose (reflecting both small intestinal and colonic permeability) (55). Other experimental animal stress models such as thermal injury or early maternal deprivation induced the development of gastric ulcers, altered gastrointestinal motility and ion secretion, and increased intestinal permeability [reviewed by Caso et al. (56)]. SGLT1 expression was markedly increased in the rat jejunum and ileum after 8 weeks of restraint stress. These findings were paralleled with an increase in intestinal lymphocytic infiltration and adrenal gland weight gain (26). The up-regulation of the SGLT1 is probably necessary to meet with the increased water, sodium, and nutrient demand, induced by chronic stress (42).

The effect of acute stress on intestinal permeability was also investigated in humans (57). In healthy volunteers subjected to a public speech test, high cortisol-responders displayed increased intestinal permeability as measured by the lactulose–mannitol ratio. Exogenous CRH administration also increased intestinal permeability, yet the CRH-induced hyper-permeability could be suppressed by the mast cell stabilizer disodium cromoglycate. Mast cell stabilization before the public speech test also did not alter intestinal permeability, however, it should be noted that in this experiment, a control group was not included. Nevertheless, these results identify CRH as an important factor in the stress-induced alterations of the intestinal barrier function. These alterations seemed to be mediated by intestinal mast cells that upon activation secrete pro-inflammatory mediators like IFN-γ and TNF-α. A variety of pro-inflammatory cytokines increases epithelial and endothelial paracellular permeability by modulating the structure of the tight junction and by inducing cytoskeletal disruptions via activation of MLCK (32, 34, 58) (Figure 1). For example, IFN-γ increased epithelial permeability of T84 monolayers to large molecules (10 kDa). Interestingly, the IFN-γ-induced increase in permeability also up-regulated the passage of FITC-labeled-endotoxin by 10-fold (59).

## Neuroendocrine–Immune Interactions

The complex neuroendocrine–immune interactions are evidenced by the fact that emotional stressors influence the immune response and that pure immunological stimuli impact on cognitive performance (60). Inflammatory mediators activate the HPA-axis with the purpose to provoke disease behavior and redirect energy-rich nutrients toward the immune system (61). Cytokines have been shown to increase nutrient availability to meet with the inflammation-dependent increased metabolic demand. For example, the cytokine IL-1α increased whole body glucose metabolism on a central level (62) and cytokines like IL-6, TNF-α, IL-1, and IFN independently evoke a HPA-axis response (63–65). Immune mediators can communicate with the brain via several pathways. By stimulating afferent sensory nerve fibers, by entering the brain via the circumventricular organs or by binding to cerebral blood vessel endothelium, immune mediators effectively redirect energy-rich substrates toward the immune system (41, 42).

Besides inflammatory cytokines, prostaglandins synthesized via the cyclooxygenase system play a central role in inflammation and HPA-axis activation. Zimomra et al. (65) demonstrated that in rats the initial activation of the HPA-axis by LPS is mediated by prostaglandins, like PGE2, while inflammatory cytokines maintain corticosterone levels at later time-points. In this study, it was suggested that prostaglandins stimulated corticosterone release in a direct manner, since the peak in circulating corticosterone levels was observed long before the peak in circulating ACTH. This idea was confirmed by a study in rodents, showing that PGE2 directly stimulated the release of glucocorticoids from the adrenal gland (66). In human adrenal cells expressing TLR2 and TLR4, LPS stimulation resulted in the release of cortisol. This effect was mediated by PGE2, since inhibition of cyclooxygenase-2 attenuated cortisol release (67).

As indicated, TLR4 activation stimulates the release of PGE2 by immune cells, adipocytes, endothelial, epithelial, and probably also adrenal cells (68), inducing the peripheral release of glucocorticoids from the adrenal gland (66). PGE2 also activates glucocorticoid production through activation of the HPA-axis at the level of the hypothalamus and the pituitary (69). Macrophages, homing in blood vessels in the cranium, are directly activated by danger signals such as LPS. Activation of these special macrophages induces the production of PGE2 which directly stimulates the paraventricular nucleus of the hypothalamus, leading to higher production of glucocorticoids, which should probably protect against possible inflammation of the brain (69).

## Acute Stress Stimulates Pro-Inflammatory Pathways by Increasing Intestinal Permeability

Acute stress modulates the immune response and changes immune cell distribution. These neuroendocrine effects on the immune system are mediated by stress-hormones released from the adrenal gland, by direct innervation of sympathetic nerve fibers into lymphoid organs and by stress hormone receptors expressed on immune cells, like glucocorticoid receptors (GRs) and α- and β-adrenergic receptors (70–72). It has been suggested that by mobilizing immune cells, the stress response, also known as the “fight–flight reaction,” prepares the immune system for oncoming challenges (70).

**Figure 2:**
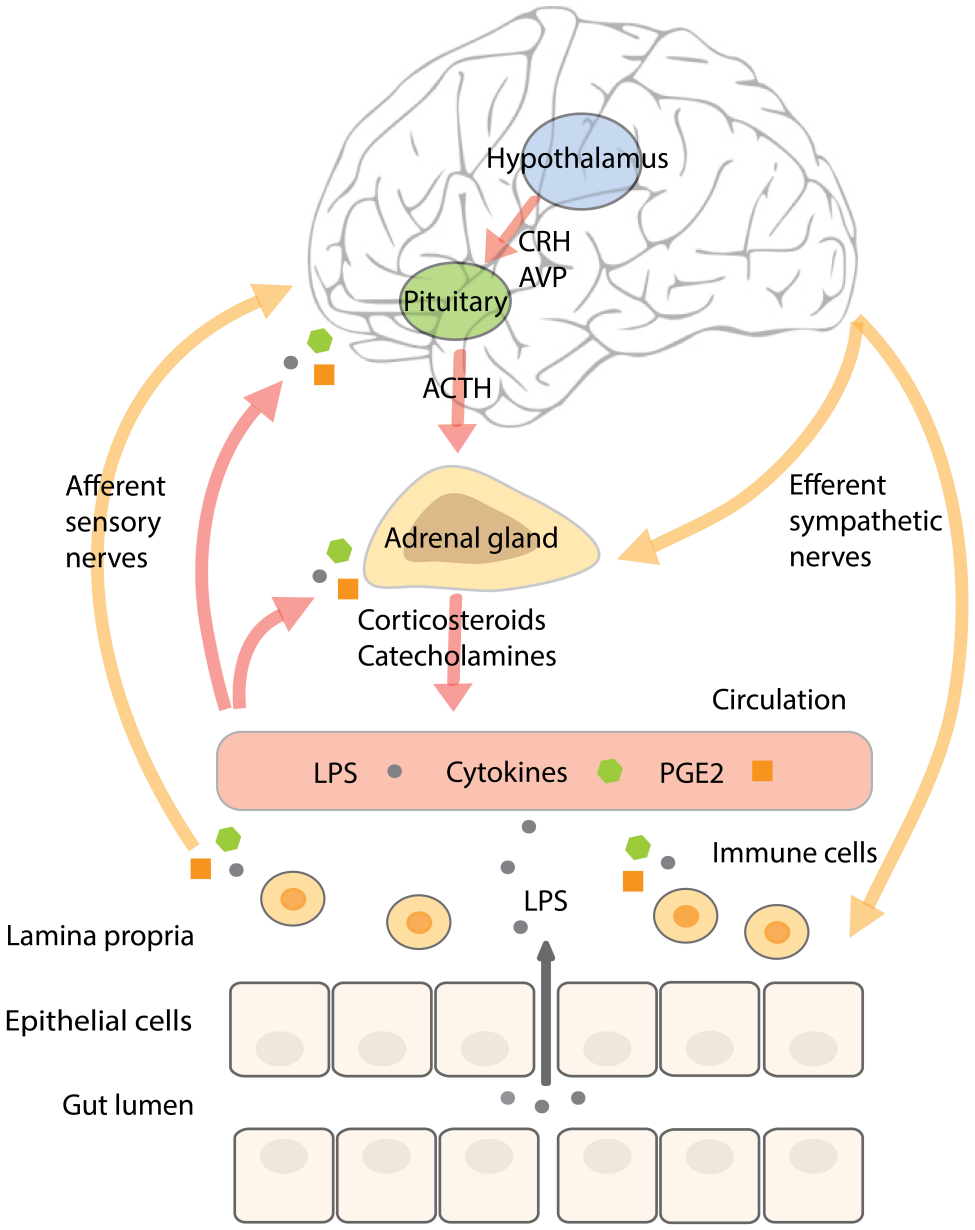
**The complex neuroendocrine–immune interactions and their relation to gut barrier function**. Stressors, including inflammatory mediators, activate the SNS and HPA-axis. Activation of the HPA-axis stimulates neurons in the paraventricular nucleus of the hypothalamus to secrete CRH and AVP that trigger the release of ACTH from the anterior pituitary, resulting in the secretion of corticosteroids from the adrenal cortex. CRH has been shown to affect intestinal permeability. SNS activation results in the release of catecholamines from the adrenal medulla. The intestinal wall is innervated by adrenergic sympathetic nerve fibers that upon stimulation increase water, sodium, and glucose absorption, paralleled by increased intestinal permeability. The resulting increase in translocation of endotoxin across the intestinal barrier can stimulate immune cells in the underlying lamina propria to secrete pro-inflammatory cytokines and prostaglandins like PGE2. Inflammatory mediators communicate with the brain by stimulating afferent sensory nerve fibers, by entering the brain via the circumventricular organs or by binding to cerebral blood vessel endothelium. Continuous stress-induced impairment of the intestinal barrier creates a vicious circle whereby inflammatory cytokines will persistently activate the SNS and HPA-axis resulting in barrier disruption, increased endotoxin translocation, and a pro-inflammatory state.

In addition, acute stress increases circulating pro-inflammatory mediators (73–75). In subjects exposed to acute stress, NF-κB was up-regulated in peripheral blood mononuclear cells in parallel with elevated levels of circulating catecholamines and glucocorticoids (76). Until now, it is not completely understood what causes this pro-inflammatory response. Glucocorticoids mostly have an inhibitory effect on inflammatory pathways and catecholamines a rather modulating than activating influence on the immune system (71, 72, 77), however, it has been shown that activation of the β-adrenergic receptor by noradrenalin (but not adrenalin) increased NF-κB binding to DNA in monocytes *in vitro* (76). A recent study in rodents showed that acute stress-induced neuro-inflammation could be prevented by a pre-stress treatment with antibiotics or an inhibitor of MLCK. In addition, these treatments prevented stress-induced hyper-permeability and endotoxemia, indicating that it is not the stress-factor itself producing a pro-inflammatory response of the immune system, but the fact that stress increases barrier permeability and the translocation of endotoxin. Pre-stress probiotic treatment with *Lactobacillus farciminis* had similar effects, which could be explained by its ability to enhance intestinal barrier function (78). In agreement with these results, it could be hypothesized that the (short-lasting) pro-inflammatory activity in humans observed during acute stress is initiated by a stress-induced increase in intestinal permeability, mediated by the SNS and components of the HPA-axis, and resulting in higher levels of translocating endotoxin interacting with TLRs on immune cells, adipocytes, and epithelial cells. A schematic overview of the complex neuroendocrine–immune interactions and their relation to gut barrier function are displayed in Figure 2.

## Chronic Stress Dysregulates the HPA-Axis and Changes Immune Function

Chronic psychological stress is known to dysregulate the immune system. These alterations are accompanied by low-grade inflammation, delayed wound healing, and increased susceptibility to infectious diseases (79). Chronic stress leads to hypercortisolemia (77), long-term permeability of barriers, endotoxemia, and low-grade inflammation (our hypothesis and theory). Normally, the release of glucocorticoids puts a limit on the maximum activity of the immune system; however, chronic HPA-axis stimulation can result in glucocorticoid resistance at the level of the immune system, making it insensitive to its inhibitory and modulatory actions (2). This process is observed in several conditions (including conditions related to psychosocial stress), whereby immune cells from patients are less responsive to the inhibitory actions of glucocorticoids on cytokine release and cell proliferation after stimulation *in vitro* (80–83). In addition, chronic stress induces a shift in the production of type 1 cytokines toward type 2 cytokine production. It can be deducted that by this mechanism, the part of the immune system involved in the clearance of extracellular bacteria and bacterial toxins (the type 2 response) is prevented from being suppressed and protection against ongoing microbial infiltration (endotoxemia) is guaranteed, while the type 1 response, involved in clearance of intracellular pathogens (like viruses) is inhibited (71, 84).

## Life-Style-Related Factors Induce Endotoxemia

The fact that stress increases barrier permeability and thereby enhances the availability of water, sodium, and nutrients, makes sense from an evolutionary perspective. However, the question arises if the accompanied translocation of bacteria and their toxins should also be considered beneficial for the host. We speculate that when the composition of the microbiota is physiological, and barrier opening is short-lasting, acute stress will not produce low-grade inflammation. However, modern people suffer from new multi-factorial stressors, such as chronic psychosocial stress and the consumption of a “Western diet,” which constantly challenge the stress-axis, alter microbiota composition, and thereby compromise intestinal barrier function. This next section discusses how modern life-style factors impact the gut–brain–immune-axis and promote endotoxemia, low-grade inflammation, and its related diseases.

### Gut-microbiota modulate stress-axis and influence gut barrier function

Large differences in the composition of the gut-microbiota and an overall reduction in microbial diversity are observed in Western populations when compared to traditional Hunter-gatherers or people from rural Africa (85, 86). These environment and diet-induced changes in gut-microbiota have been connected to an increased susceptibility to chronic diseases, like IBD, obesity, and type 1 and type 2 diabetes (87). The gut-microbiota influences inflammatory (88) and metabolic processes (89) and has been shown to influence the development of the HPA-axis and immune system (90, 91). For example, exposure to LPS during developmental periods can exaggerate the HPA-axis and immune response to stress (92, 93), but also the absence of bacteria can induce these effects. Animals raised in germ-free environments showed an exaggerated HPA-axis response, which was normalized by colonization with fecal matter from specifically germ-free animals or by the administration of the Gram-positive *Bifidobacterium infantis* (94). Vice versa, exposure to social stress changed the composition of the gut-microbiota in mice (95, 96) and prenatal stress altered the microbiome in rhesus monkeys by reducing the overall numbers of the Gram-positive *Bifidobacteria* and *Lactobacilli* (97), indicating that chronic stress affected the composition of the gut microbiome. Stress influences gut motility, secretions, and mucin production, thereby altering the habitat of resident bacteria, promoting changes in the composition of the gut microbiome (98), and allowing the growth of pathogenic bacteria (99).

Increasing evidence supports an important role for microbiota on the homeostasis of the intestinal barrier. Certain strains of the Gram-positive *Lactobacilli* decreased intestinal permeability in several animal and human disease models (78, 100). *B. infantis* reduced intestinal permeability (as assessed by 70-kDa fluorescein isothiocyanate–dextran transmucosal flux) and ameliorated symptoms in a neonatal necrotizing enterocolitis mouse model (101). Further evidence indicating the influence of the gut-microbiota on intestinal permeability was presented in detoxifying alcoholic-dependent subjects: lower levels of *Ruminococcaceae* and higher abundance of *Lachnospiraceae* (*Dorea*) and *Blautia* were associated with increased intestinal permeability (102). In addition, higher levels of certain pathogenic bacteria can increase intestinal permeability by disrupting the epithelial barrier and triggering cell death and inflammation. These bacteria have the ability to bind and/or translocate through endothelial and microfold cells and have been shown to secrete toxins or other effector molecules via specialized secretion systems. Although the exact mechanisms are not well described, most pathogenic gut bacteria including *Escherichia coli*, *Helicobacter pylori*, *Staphylococcus aureus*, *Cholera Pseudomonas fluorescens*, *Pseudomonas aeruginosa*, *Yersinia enterocolitica*, *Campylobacter jejuni*, and *Salmonella typhimurium* alter paracellular permeability by disassembling tight junctions and generating cytoskeleton changes by increasing inflammation [reviewed by Barreau et al. (103)]. As an example, a strain of *E. coli*, normally present in the human gut, induced focal leaks in colonic epithelial monolayers and in rat distal colon by using α-hemolysin, allowing for its paracellular translocation across the epithelial layer (104).

### High-caloric and high-fat diets induce inflammation and increase circulating endotoxin levels

Compared to healthy individuals, patients suffering from obesity have higher circulating endotoxin levels together with greater levels of circulating pro-inflammatory cytokines and insulin resistance (105). Food intake can produce post-prandial immune activation and elevate endotoxin levels when a meal is high in calories (106) or has a high fat content (6, 107–109).

Rodents fed a 4-week high-fat diet (72% fat) showed a constant elevation in circulating endotoxin levels, while in control animals, endotoxin levels only increased during feeding hours. Furthermore, a high-fat diet produced fasting glycemia, insulin resistance, general weight gain, and weight gain of the liver and visceral and subcutaneous adipose tissue. In addition, adipose tissue F4/80-positive cells (indicating the infiltration of macrophages), markers of inflammation, and liver triglyceride content were increased. Interestingly, almost similar effects were observed in mice subcutaneously infused with LPS (resulting in similar circulating LPS levels as observed in the high-fat fed mice). These effects were mediated by TLR4, since mice lacking CD14, which is important for the recognition of LPS to this receptor, showed a delayed response to a high-fat diet or LPS injections (107).

In healthy humans, a 910 calories high-fat and high-carbohydrate meal resulted in increased circulating endotoxin levels and elevated levels of LBP in parallel with higher inflammatory markers and increased protein expression of TLR2 and TLR4 in isolated leukocytes. A meal high in fruits and fiber did not induce these effects (108). Plasma endotoxin levels, pro-inflammatory markers, and leukocyte TLR4 expression increased after the intake of cream (300 calories), while the intake of 300 calories of glucose resulted only in a pro-inflammatory response and the intake of orange juice and water showed none of these effects (110). In healthy individuals, plasma endotoxin levels increased about 50% after the intake of a high-fat meal (900 calories) (6) and 4 weeks consumption of a Western-style diet raised plasma endotoxin activity levels by 71% (111).

How exactly the intake of a high-caloric meal increases circulating endotoxin levels is still unclear but has been explained by several mechanisms [reviewed by Kelly et al. (112)]. One of these suggested mechanisms is that the introduction of a high-fat diet modulates the expression of genes involved in the barrier function in epithelial cells, thereby directly compromising the integrity of the tight junction (113). Another explanation could be that a high-caloric/high-fat meal induces high levels of insulin and leptin, hormones that directly activate the SNS (114, 115). Moreover, insulin enhances SGLT1-mediated glucose absorption (116). Activation of the SGLT1 and the SNS leads to increased permeability of the gut barrier, which may induce the observed post-prandial endotoxemia (our hypothesis and theory).

### Gliadin compromises the integrity of tight junctions

The intake of wheat and other cereal grains has been implicated in the development of inflammation-related diseases, by inducing inflammation and increasing intestinal permeability (40). Gliadin, a component of gluten, has been demonstrated to increase permeability in human Caco-2 intestinal epithelial cells by reorganizing actin filaments and altering expression of junctional complex proteins (117). Several studies by the group of Fasano et al. showed that the binding of gliadin to the chemokine receptor CXCR3 on epithelial IEC-6 and Caco-2 cells releases zonulin, a protein that directly compromises the integrity of the junctional complex (118, 119).

### Alcohol consumption increases intestinal permeability

Alcohol consumption is an important risk factor for disease and is one of the major causes of chronic liver disease. Increased intestinal permeability has been observed during chronic alcohol consumption. In an animal model of chronic alcoholic liver disease, alcohol feeding for 8 weeks increased intestinal permeability (120). In humans, alcohol-dependence induced changes in the gut-microbiota composition that were associated with increased intestinal permeability (102). Furthermore, increased intestinal permeability and higher circulating endotoxin levels were observed in patients with chronic alcohol abuse (121–123).

### Exercise-induced heat-stress increases intestinal permeability

Exercise increases body temperature, reduces intestinal blood flow (reallocated to the muscles and cardiac system), and increases intestinal permeability by activating the SNS and HPA-axis. Already in 1992, Oktedalen et al. (124) showed that marathon runners displayed a significant increase in intestinal permeability. In addition, studies have indicated that strenuous exercise induced higher circulating endotoxin levels and activated the immune system (125–128). Further evidence of exercise- and heat-induced increased intestinal permeability, leading to gastrointestinal complaints in people engaging in physical activity, has been recently reviewed (129).

## Endotoxemia is Associated with Diseases Related to Chronic Inflammation

Multiple human studies have emerged that find associations with NCDs and markers of endotoxemia. Even aging, associated with higher sympathetic nerve activity (130) and higher circulating inflammatory mediators like IL-6, has been linked to higher plasma concentrations of LPS and LBP (131). In further support of our theory, in this section, an overview is given of human studies finding changes in levels of endotoxin or endotoxin-related markers in NCDs (Table 1).

**Table 1:** **Associations found between markers of endotoxemia and disease**.

Reference	Disease	Marker(s) of endotoxemia	Effect
(132)	Metabolic syndrome	Serum LPS	LPS levels correlated positively with symptoms of metabolic syndrome
(133)	Obesity-related insulin resistance	Serum LBP	LBP levels increased
(134)	Psoriasis/metabolic syndrome	Serum LBP	LBP levels only increased in psoriasis patients with metabolic syndrome
(135)	Obesity	Plasma LBP	LBP levels increased
(136)	NAFLD	Plasma LPS	LPS levels increased
(14)	NAFLD	Serum LPS	LPS levels increased
(137)	Obesity/NAFLD	Plasma LBP	LBP levels increased
(122)	Liver disease	Plasma LPS	LPS levels increased
(12)	Type 2 diabetes	Serum LPS	LPS levels increased
(138)	Type 2 diabetes	Plasma LPS	LPS levels increased
(139)	Type 2 diabetes	Serum LPS	LPS levels increased
(140)	Diabetes	Serum LPS	LPS levels increased
(141)	Type 2 diabetes, impaired glucose tolerance	Serum LPS	LPS levels increased
(142)	Cardiovascular diseases	Serum LPS, serum IgA/IgG against oral bacteria	LPS levels increased, no differences in IgA/IgG levels
(143)	Coronary artery disease	Plasma LBP	LBP levels increased
(144)	Coronary artery disease	Serum LBP	LBP levels increased
(145)	Arteriosclerosis	Serum LBP	LBP levels increased
(146)	Arteriosclerosis	Plasma LBP	LBP levels increased
(11)	Chronic heart failure (edematous)	Plasma LPS	LPS levels increased in edematous vs. non-edematous patients. No differences between all patients vs. controls
(147)	Chronic heart disease (edematous)	Plasma LPS	LPS levels increased in edematous vs. non-edematous patients
(148)	IBD	Serum LPS, LBP, sCD14	LPS, LBP, sCD14 levels increased
(149)	IBD	Plasma LPS, LBP, sCD15, endoCAbs	No differences in levels of LPS, sCD14, and endoCAbs. LBP levels increased
(150)	IBD	Plasma LPS, endoCAbs	LPS and endoCAbs levels increased with disease severity
(151)	Parkinson’s disease	Serum LBP, *E. coli* LPS infiltration in intestinal tissue	LBP levels decreased, increased LPS infiltration in intestinal tissue
(13)	Autism	Serum LPS, sCD14	LPS levels increased, no differences in sCD14 levels
(152)	Sporadic amyotrophic lateral sclerosis, Alzheimer’s disease	Plasma LPS	LPS levels increased
(9)	Depression	Serum IgA/IgM against intestinal bacteria	IgA/IgM levels increased
(10)	Chronic fatigue syndrome	Serum IgA/IgM against intestinal bacteria	IgA/IgM levels increased
(153)	Alzheimer’s disease	Serum IgG against oral bacteria	IgG levels increased

### Metabolic syndrome

Metabolic syndrome is accompanied by an increased risk for NAFLD, obesity, type 2 diabetes, and cardiovascular diseases. All of these conditions are related to and even predicted by increased sympathetic nerve activity (154) and a dysregulated HPA-axis (155). Higher circulating endotoxin and LBP levels are associated with risk factors of the metabolic syndrome, like insulin resistance, obesity, dyslipidemia, and chronic inflammation (132–135). Patients suffering from NAFLD exhibited significantly higher serum endotoxin levels in contrast to healthy controls (14). Farhardi et al. (136) indicated that elevated plasma endotoxin levels in these patients were related to an impaired intestinal barrier function, because, only in the patient group, the intake of a permeability stressor (aspirin) increased the 0–24 h urinary excretion of sucralose (a marker of whole-gut permeability). Furthermore, augmented plasma LBP levels in concert with increased plasma levels of TNF-α were observed in obese NAFLD patients compared to healthy controls (137).

Elevated circulating levels of endotoxin and LBP were detected in type 2 diabetics (12, 133, 138–140). Compared to healthy controls, obese individuals and type 2 diabetics showed higher endotoxin levels after the intake of a high-fat meal. Increased endotoxin levels were observed in all challenged individuals, yet higher endotoxin levels were seen in individuals suffering from metabolic illnesses, suggesting an increased intestinal permeability in these patients (141). This was further indicated by a recent study showing that increased serum levels of endotoxin, IL-6, and TNF-α were found in type 2 diabetic patients compared to healthy individuals. The level of endotoxin was positively related to zonulin, a marker for intestinal permeability (12).

A large cohort of patients with coronary artery disease identified increased serum LBP levels to be associated with total and cardiovascular mortality (144). Moreover, circulating LBP levels were associated with carotid intima media thickness (a marker of atherosclerosis), obesity, insulin resistance, and high-sensitive CRP (145).

Patients suffering from chronic heart failure with aggravated renal function displayed increased circulating endotoxin levels and an impairment of the intestinal barrier (11). Wiedermann et al. (146) showed that subjects with the highest levels of circulating endotoxin (90th percentile) had a threefold increased risk of incident atherosclerosis. Higher serum endotoxin and pro-inflammatory cytokine concentrations were seen in patients with edematous chronic heart disease compared to stable patients and healthy controls. Intriguingly, after short-term diuretic treatment, circulating endotoxin concentrations decreased in edematous patients (147). Diuretic treatment [like angiotensin-converting enzyme (ACE) inhibitors] ameliorated intestinal inflammation, perhaps by impacting on intestinal permeability through interference with the renin–angiotensin–aldosterone system. Several components of this system (renin, ACE, and angiotensin II) have been shown to stimulate pro-inflammatory pathways (44, 156).

### Inflammatory bowel disease

Ulcerative colitis and Crohn’s disease are intestinal inflammatory disorders, also known as IBD, which have been causally linked to chronic psychological stress (157), altered immune function, changes in the gut-microbiota, increased intestinal permeability, and endotoxemia (158). For example, increased plasma endotoxin and LBP levels were measured in both patient groups, but were more pronounced in patients with active disease compared to inactive disease and were associated with disease severity (148). In addition, detectable plasma endotoxin levels and higher plasma levels of LBP were more frequently observed in IBD patients compared to controls (15, 149) and were correlated with disease severity and circulating TNF-α levels (150).

### Psychiatric diseases

Over the last decade, the role of the gut–brain axis has emerged as an important mediator in the development of psychiatric and mood disorders (159). Moreover, higher endotoxin levels and intestinal barrier dysfunction are observed in several of these conditions. For example, Parkinson’s patients exhibited increased total intestinal permeability and a more intense staining for *E. coli* LPS and oxidative stress markers in intestinal sigmoid mucosa samples. However, in these patients, endotoxin levels resembled control samples and serum LBP concentrations were lower compared to healthy individuals (151). Higher serum endotoxin levels are associated with severe autism, sporadic amyotrophic lateral sclerosis, and Alzheimer’s disease (13, 152). Furthermore, increased IgA and IgM responses against LPS of commensal bacteria were seen in chronic fatigue syndrome (10) and depression (9). Intriguingly, chronic oral infection of periodontitis was associated with Alzheimer’s disease where higher antibody levels against oral pathogens were observed years before the onset of symptoms in people suffering from Alzheimer’s disease (153), suggesting there was an increased translocation of bacteria and/or bacterial toxins from the mouth into the bloodstream.

## Conclusion

Chronic low-grade inflammation is an eminent feature of NCDs. In addition, many studies report increased circulating endotoxin levels and increased gut permeability in patients suffering from these conditions. As reviewed in this paper, stress-induced increases in intestinal permeability, in combination with modern life-style factors, raise the possibility of translocation of bacteria and/or their toxins across the more permeable gut barrier. The resulting, long lasting, endotoxemia should be considered much more than just a risk factor for chronic disease; it could be a cause. Notwithstanding the fact that the exact origin and sequence of events involved in development of NCDs remain to be unsolved, evidence indicates that a disrupted barrier function in parallel with elevated circulating endotoxin levels may underlie disease onset and progression. For this reason, therapies aimed at restoring intestinal barrier function, life-style changes, and stress management should be considered important strategies in preventing and attenuating the pro-inflammatory state observed in NCDs.

## Conflict of Interest Statement

The authors declare that the research was conducted in the absence of any commercial or financial relationships that could be construed as a potential conflict of interest.
